# Supplementary material for: An optimized retroviral toolbox for overexpression and genetic perturbation of primary lymphocytes
Source: Biol Open. 2022 Mar 1;11(2):bio059032. doi: 10.1242/bio.059032 (PMC8905627; doi:10.1242/bio.059032)
Supplement: Supplementary information [file biolopen-11-059032-s1.pdf]

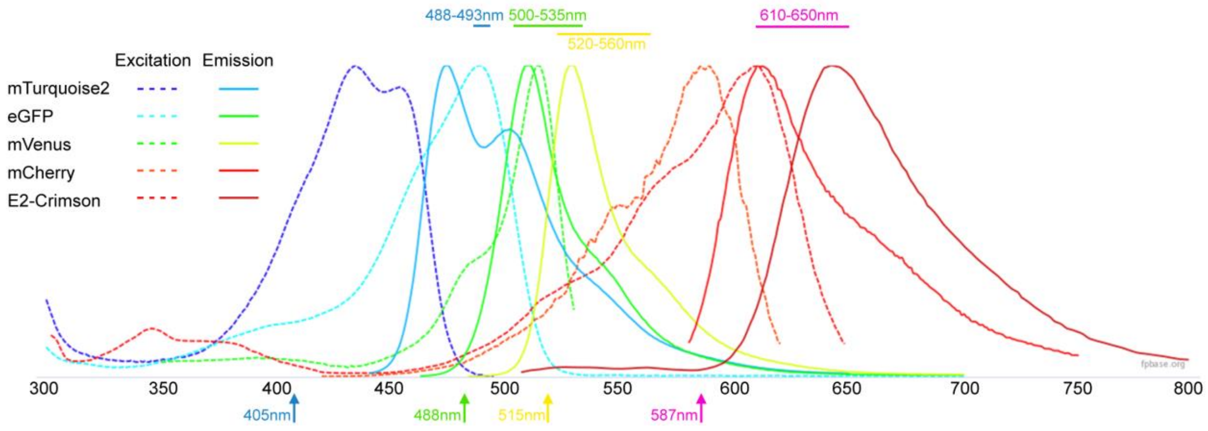

Fig. S1. Excitation and emission spectra of the used fluorescent proteins.

The X-axis depicts the wavelength in nm; Y-axis depicts relative fluorescent intensity for each fluorophore. Primary investigations with E2-Crimson were not promising and this protein was therefore not included in the vector set. Not that excitation and emission spectra of eGFP and mVenus are quite close and therefore these proteins should only be used together with that knowledge considered. Colored arrows at the bottom indicate used excitation wavelengths. Bars and text at the top indicate acquisition wavelengths used for mTQ2, GFP, mVenus and mCherry respectively. Figure was created with fpbase.org spectra-viewer tool (Lambert, 2019).

Biology Open • Supplementary information

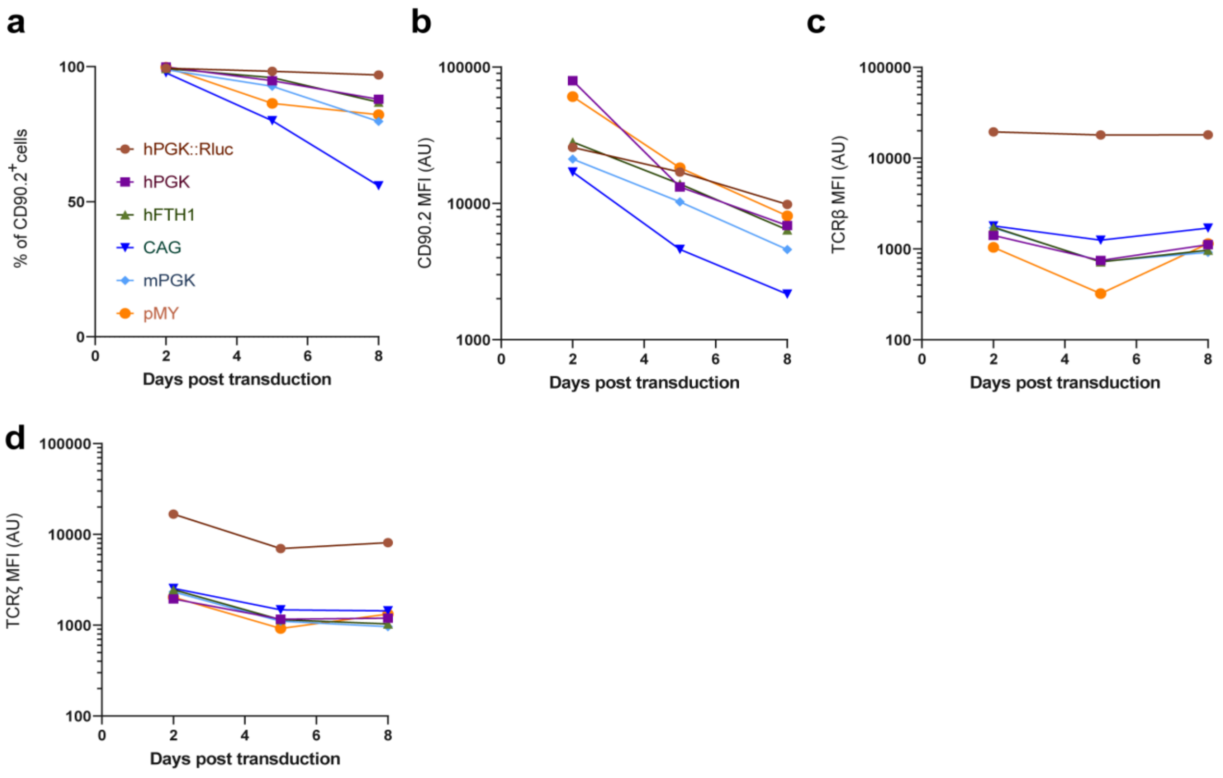

Fig. S2. Vector stability and expression *in vitro*, without the addition of IL-7. The experiment in Figure 4c-f was repeated, but cells were maintained constantly on IL-2 instead of changing to IL-7 supplementation 5 days post transduction. Comparable results regarding the proportion of $CD90.2^{+}$ cells (a) and the expression levels of the CD90.2 surface marker (b), TCRβ 9c) and TCRζ within the $CD90.2^{+}$ population were obtained, except for the steep decline in the percentage of $CD90.2^{+}$ cells observed for cells transduced with pMY observed before. Note that without the addition of IL-7 the cells stop proliferating strongly after 5 days post-transduction and therefore the experiment was terminated at day8.

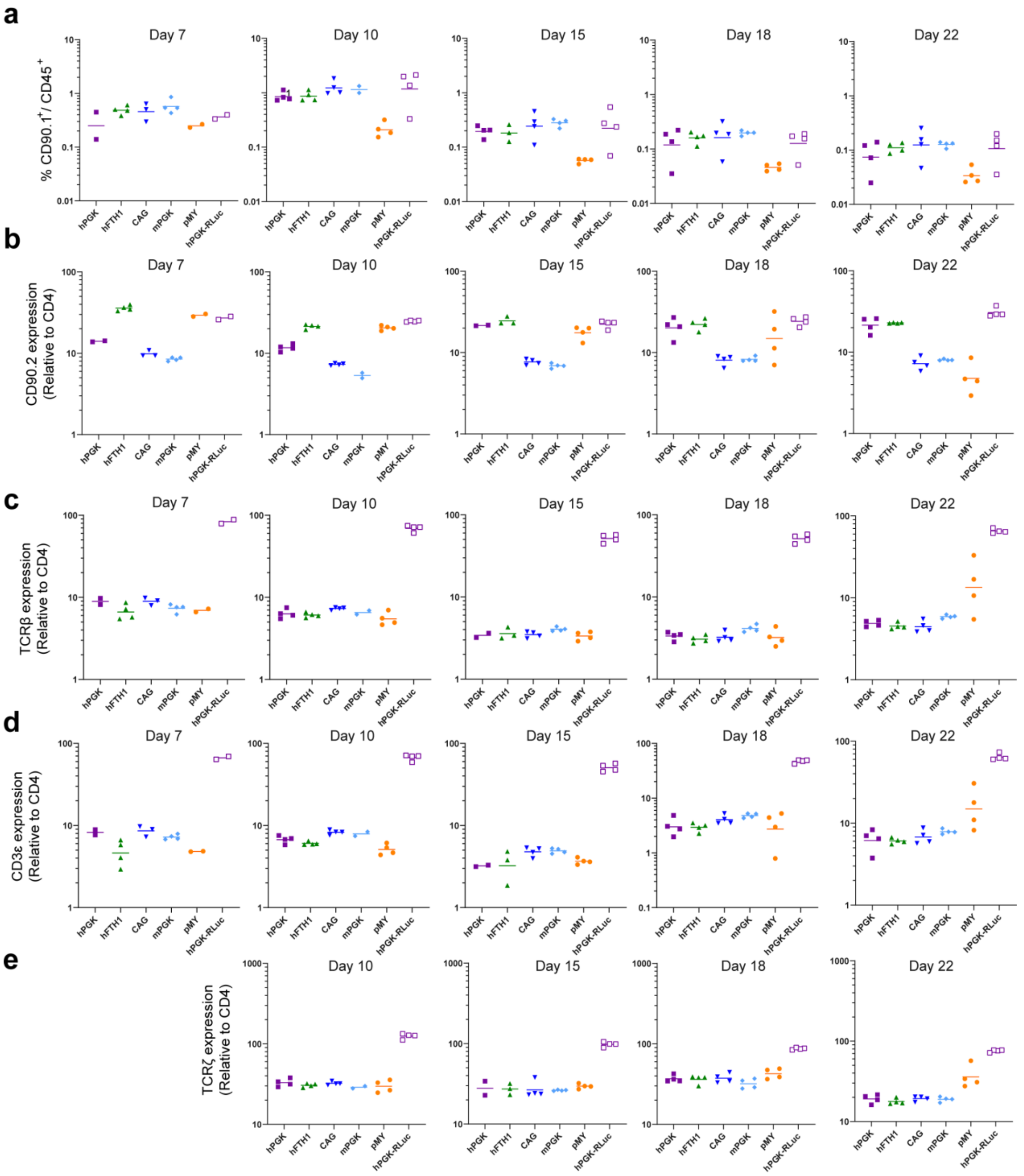

Fig. S3. Individual values of *in vivo* vector validation in blood. Individual values corresponding to the data of figure 5a-e. Cells obtained from tail-vein bleeds at the indicated timepoints were assessed by flow cytometry. Bars depict the mean.

Biology Open • Supplementary information

Table S1. shRNA-miRs used in this study.

The antagomir target sequences were derived from the genetic perturbation platform of the Broad Institute (https://portals.broadinstitute.org/gpp/public/). Bold sequences were selected and used throughout the manuscript.

Target	shRNA-MiR code	Target Sequence
**CD247/TCRζ-A**	TRCN0000068158	CCTCTTCATCTACGGAGTCAT
CD247/TCRζ-B	TRCN0000068159	GCTAGATGGAATCCTCTTCAT
CD247/TCRζ-C	TRCN0000068160	CGTATACAATGCACTGCAGAA
CD247/TCRζ-D	TRCN0000068161	CTCTACAATGAGCTCAATCTA
CD247/TCRζ-E	TRCN0000068162	CCAATCCTGTGCCAGCGTCTT
**Renilla Firefly luciferase**	SHC007	CCGCTGAGTACTTCGAAATGTC

Table S2. Primers used in this study.

Primer name	Primer sequence 5' → 3'
**hFTH1prom_Pac_Fwd**	GCAGAACCTTAATTAATCCGCCAGAGCGCGCGAG
**hFTH1prom_Bam_Rev**	ATGCATCCGGATCCGGCGGCGACTAAGGAGAGGC
**mPGK_prom_Pac_Fwd**	GCAGAACCTTAATTAAAAATTCTACCGGGTAGGGGAGGCGCTTTTC
**mPGK_prom_Bam_Rev**	ATGCATCCGGATCCGTCGAAAGGCCCGGAGATGAGGAAGA
**hPGK_Fwd (PacI)**	GCAGAACCTTAATTAACGGGGTTGGGGTTGCGCCTTT
**hPGK_Rev (BamHI)**	ATGCATCCGGATCCCTGGGGAGAGAGGTCGGTGATTCG
**CMV_prom_Pac_Fwd**	GCAGAACCTTAATTAAGACATTGATTATTGACTAGTTATTAATAGTAATCAATTACGGGGTCAT
**CMV_prom_Bam_Rev**	ATGCATCCGGATCCAGCTCTGCTTATATAGACCTCCCACCGTA
**miR30_Hind_Fwd**	GCAGAACCAAGCTTTGTTTGAATGAGGCTTCAGTACTTTACAGAAT

Biology Open • Supplementary information

**miR30_Cla_Rev**	ATGCATCCATCGATAAAGTGATTTAATTTATACCATTTTAATT CAGCTTTGT
**miRE-Xho-Fwd**	TACAAATACTCGAGAAGGTATATTGCTGTTGACAGTGAGCG
**miRE-Eco-Rev**	ACTTAGAAGAATTCTAGCCCCTTGAAGTCCGAGGCAGTAGGC
**Ly6Gopt_SalP2A_Fwd**	CAGAACCACGCGTGGCCTGGCATGCCGCGACGTCGACGCCAC AAACTTCAGCCTGCTGAGACAGGCTGGCGACGTGGAAGAGAA TCCTGGACCTATGGACACCTGTCATATCGCCAAGAG
**CD90.2_SalP2A_Fwd**	GCAGAACCACGCGTGGCCTGGCATGCCGCGACGTCGACGCCA CAAACTTCAGCCTGCTGAGACAGGCTGGCGACGTGGAAGAGA ATCCTGGACCTATGAACCCCGCCATCTCTGTG
**Ly6Gopt_Sbf_Rev**	ATGCATCCCCTGCAGGTCACAGGAAGGTCTGCAGCAGAA
**CD90.2_Sbf_Rev**	ATGCATCCCCTGCAGGCTACAGAGAGATGAAGTCCAGGGC
**P2A_SalI_Fwd**	GCAGAACCGTCGACGCCACGAACTTCTCTCTGTTAAGACAA
**GFP_Sbf_Rev**	ATGCATCCCCTGCAGGTTACTTGTACAGCTCGTCCATGCCG
**BlastR_SalP2A_Fwd**	CAGAACCGTCGACGCCACAAACTTCAGCCTGCTGAGACAGGC TGGCGACGTGGAAGAGAATCCTGGACCTATGAAAACATTTAA CATTTCTCAACAAGATCTAGAATTAGTAGAAGTAGC
**BlastR_DYK_Sbf_Rev**	ATGCATCCCCTGCAGGTCATTTGTCGTCGTCGTCCTTGTAGTC GCCGCTGCCATTTCGGGTATATTTGAGTGGAATGAGTTCTTCA ATCG
**PuroR_SalP2A_Fwd**	GCAGAACCACGCGTGGCCTGGCATGCCGCGACGTCGACGCCA CAAACTTCAGCCTGCTGAGACAGGCTGGCGACGTGGAAGAGA ATCCTGGACCTATGACCGAGTACAAGCCCACG
**PuroR_HA_Sbf_Rev**	ATGCATCCCCTGCAGGTCAGGCGTAATCAGGCACATCGTAAG GGTAGCCGCTGCCGGCACCGGGCTTGCGGG
**mCherry_Sal_GSG_Fw**	GCAGAACCGTCGACGGATCTGGCATGGTGAGCAAGGGCGAG GA
**mCherry_Hind_Rv**	ATGCATCCAAGCTTTTACTTGTACAGCTCGTCCATGCC
**Lyz2_MluI_Fwd**	GCAGAACCACGCGTGCCACCATGAAGACTCTCCTGACTCTGG GACT
**Lyz2_GSG_SalI_Rev**	ATGCATCCGTCGACGCCAGATCCGACTCCGCAGTTCCGAATAT ACTGG
**EFS_Pac_Fwd**	GCAGAACCTTAATTAAGGCTCCGGTGCCCGTCAGT
**EFS_Bam_Rv**	ATGCATCCGGATCCCCTGTGTTCTGGCGGCAAACC
**SHC007_shRNAmiR_temp**	TGCTGTTGACAGTGAGCGCCGCTGAGTACTTCGAAATGTCTAG TGAAGCCACAGATGTAGACATTTCGAAGTACTCAGCGTTGCCT ACTGCCTCGGA
**Cd247A_miR_temp**	TGCTGTTGACAGTGAGCGCCCTCTTCATCTACGGAGTCATTAG TGAAGCCACAGATGTAATGACTCCGTAGATGAAGAGGATGCC TACTGCCTCGGA

Biology Open • Supplementary information

Table S3. Antibody combinations used for flow cytometry staining.

Details on the individual antibodies can be found in Supplemental table 4. *: Intracellular stain performed after fixation and permeabilization. #: Marker specific staining was used where opportune.

	Transfection (Panel 1)	MACS (Panel 2)	*In vitro/vivo* (Panel 3)
FITC	Ly6G#	B220	CD3ε
PE		CD62L	TCRζ*
PE/Dazzle594		CD45	CD45
PerCP/eFluor710		CD11a	CD90.2
PE/Cy7		CD4	CD4
APC	CD90.2#	TCRβ	TCRβ
AlexaFluor700		CD90.1	CD90.1
eFluor 780	Viability	Viability	Viability

Table S4. Antibody clones, suppliers and dilutions used in this study.

**Name**	**reactivity**	**Supplier**	**Isotype**	**Clone**	**Catalog #**	**Dilution**
Ly-6G/Ly-6C-FITC	Mouse	eBiosci.	Rat IgG2b, κ	RB6-8C5	11-5931-82	1:400
CD90.2-APC	Mouse	BD Biosci.	Rat IgG2a, κ	53-2.1	553007	1:400
CD90.2- PerCP eFluor710	Mouse	eBiosci.	Rat IgG2b, κ	30-H12	46-0903-80	1:400
CD45R/B220-FITC	Mouse	BD Biosci.	Rat IgG2a, κ	RA3-6B2	553087	1:100
CD62L-PE	Mouse	BD Biosci.	Rat IgG2a, κ	MEL-14	553151	1:100
CD45-PE/Dazzle594	Mouse	Biolegend	Rat IgG2b, κ	30-F11	103146	1:800
CD11a-PerCP eFluor710	Mouse	eBiosci.	Rat IgG2a, κ	M17/4	46-0111-80	1:400
CD4-PE/Cy7	Mouse	Biolegend	Rat IgG2a, κ	RM4-5	100528	1:400
TCRβ- APC	Mouse	Biolegend	Hamster/IgG	H57-597	109212	1:100
TCRζ (CD247)-PE	Human/ Mouse	Biolegend	Mouse IgG1κ	6B10.2	644106	1:100
CD90.1-AlexaFluor700	Mouse	Biolegend	Mouse IgG1κ	OX-7	202528	1:400
CD3ε-FITC	Mouse	Biolegend	Hamster IgG	145-2C11	100306	1:100
Fixable viability dye eFLuor780	N/A	eBiosci.	N/A	N/A	N/A	1:1000

Biology Open • Supplementary information

Table S5. Synthesized DNA sequences.

CD3-complex optimized
notI-gamma-mfeI-T2A-delta-mluI-F2A-epsilon-SphI-E2A-zeta-SalI-P2A-Ly6G-SbfI:

GCAGAACCGCGGCCGCGCCACCATGGAACAGAGAAAGGCCTGGCCGGCCTGTTCCTG
GTTATCAGTCTGCTGCAGGGCACAGTGGCCCAGACCAACAAGGCTAAGAACCTGGTGC
AGGTGGACGGCTCTAGAGGCGACGGATCTGTGCTGCTGACATGTGGCCTGACCGACAA
GACCATCAAGTGGCTGAAGGACGGCTCCATCATCAGCCCTCTGAACGCCACCAAGAAC
ACCTGGAACCTGGGCAACAACGCCAAGGACCCCAGAGGCACCTATCAGTGCCAGGGC
GCCAAAGAGACAAGCAACCCTCTGCAGGTCTACTACAGAATGTGCGAGAACTGCATCG
AGCTGAACATCGGCACCATCAGCGGCTTCATCTTCGCCGAAGTGATCAGCATCTTCTTT
CTGGCCCTGGGCGTGTACCTGATCGCTGGACAAGATGGCGTGCGGCAGAGCAGAGCCA
GCGATAAGCAGACACTGCTGCAGAACGAGCAGCTGTACCAGCCTCTGAAGGACAGAG
AGTACGACCAGTACAGCCACCTCCAGGGCAACCAGCTGCGGAAGAAGGGATCTGGCC
AATTGGAAGGCAGAGGCTCTCTTCTTACATGCGGCGACGTCGAGGAAAACCCAGGACC
TATGGAACACTCTGGCATCCTGGCTAGCCTGATCCTGATTGCCGTTCTGCCTCAAGGCA
GCCCCTTCAAGATCCAAGTGACCGAGTACGAGGACAAGGTGTTCGTGACCTGCAACAC
CAGCGTGATGCACCTGGATGGCACCGTGGAAGGATGGTTCGCCAAGAACAAGACCCTG
AACCTCGGCAAGGGCGTGCTGGACCCTAGAGGCATCTACCTGTGTAACGGCACAGAGC
AGCTGGCCAAGGTGGTGTCTAGTGTGCAGGTCCACTATCGGATGTGTCAGAACTGCGT
GGAACTGGACAGCGGCACAATGGCCGGCGTGATCTTCATCGACCTGATCGCTACCCTG
CTGCTGGCACTGGGAGTGTATTGCTTCGCTGGCCACGAGACAGGCAGACCTAGCGGAG
CTGCTGAAGTTCAGGCCCTGCTGAAGAATGAACAGCTCTATCAGCCCCTGCGCGACAG
AGAGGATACCCAGTACTCTAGACTCGGCGGCAACTGGCCCAGAAACAAGAAATCTGGA
AGCGGCACGCGTGTCAGACAGACCCTGAACTTCGATCTGCTTAGACTGGCCGGGGACG
TCGAGTCTAATCCAGGACCAATGCGGTGGAACACCTTCTGGGGCATCCTGTGTCTGTCT
CTGCTGGCTGTGGGCACCTGTCAGGATGACGCTGAGAACATCGAGTATAAGGTGTCCA
TCTCCGGCACCAGCGTCGAGCTGACTTGTCCTCTGGACTCCGACGAGAACCTGAAGTG
GGAGAAGAACGGCCAAGAGCTGCCTCAGAAGCACGACAAGCACCTGGTGCTGCAGGA
CTTCAGCGAGGTGGAAGATAGCGGCTACTACGTGTGCTACACCCCTGCCAGCAACAAG
AACACATACCTGTACCTGAAGGCTCGCGTGTGCGAGTACTGTGTCGAGGTGGACCTGA
CAGCCGTGGCTATCATCATCATCGTGGACATCTGCATCACCCTGGGCCTGCTGATGGTC
ATCTACTACTGGTCCAAGAACCGGAAGGCCAAGGCCAAGCCTGTGACAAGAGGAACC
GGCGCTGGAAGCAGACCAAGAGGCCAGAACAAAGAAAGACCTCCTCCTGTGCCTAATC
CTGACTACGAGCCCATCCGGAAGGGCCAGAGAGATCTGTACTCTGGCCTGAACCAGAG
GGCCGTGGGTTCTGGCGCATGCCAGTGTACCAACTATGCTCTCCTGAGACTCGCAGGCG
ACGTTGAGAGTAATCCAGGGCCTATGAAGTGGAAAGTGTCTGTGCTGGCCTGCATCCT
GCATGTTCGATTCCCTGGCGCTGAGGCCCAGTCTTTTGGACTGCTGGACCCCAAGCTGT
GCTACCTGCTGGACGGCATTCTGTTTATTTATGGCGTGATCATCACCGCTCTGTACCTGC
GGGCCAAGTTCAGCAGAAGCGCTGAGACAGCTGCCAATCTGCAGGACCCTAACCAGCT
GTACAACGAGCTGAATCTGGGGCGCAGAGAAGAGTACGATGTGCTGGAAAAGAAGAG
AGCCAGAGATCCCGAGATGGGCGGCAAACAGCAGAGAAGGCGGAATCCTCAAGAAGG
CGTGTACAACGCCCTGCAGAAAGATAAGATGGCCGAGGCCTACAGCGAGATCGGCAC
AAAGGGCGAACGCAGAAGAGGCAAGGGACACGATGGACTGTACCAGGGCCTGTCCAC
AGCCACAAAGGACACATACGATGCCCTGCACATGCAGACACTGGCCCCTAGAGGCAGC
GGCGTCGACGCCACAAACTTCAGCCTGCTGAGACAGGCTGGCGACGTGGAAGAGAATC

CTGGACCTATGGACACCTGTCATATCGCCAAGAGCTGCGTGCTGATCCTGCTGGTGGTT
CTGCTGTGTGCCGAGCGAGCACAGGGACTGGAGTGCTACAACTGTATCGGCGTGCCAC
CTGAGACAAGCTGCAACACCACCACCTGTCCTTTCAGCGACGGCTTCTGTGTGGCCCTG
GAAATCGAAGTGATCGTGGACAGCCACCGCAGCAAAGTGAAGTCCAACCTGTGCCTGC
CTATCTGCCCCACCACACTGGACAACACCGAGATCACAGGCAACGCCGTGAACGTGAA
AACCTACTGCTGCAAAGAGGACCTCTGCAACGCCGCTGTTCCAACAGGCGGAAGCTCT
TGGACAATGGCTGGCGTGCTGCTGTTCAGCCTGGTGTCTGTTCTGCTGCAGACCTTCCT
GTGACCTGCAGGGGATGCAT

hPGK:

TTAATTAACGGGGTTGGGGTTGCGCCTTTTCCAAGGCAGCCCTGGGTTTGCGCAGGGAC
GCGGCTGCTCTGGGCGTGGTTCCGGGAAACGCAGCGGCGCCGACCCTGGGTCTCGCAC
ATTCTTCACGTCCGTTCGCAGCGTCACCCGGATCTTCGCCGCTACCCTTGTGGGCCCCC
CGGCGACGCTTCCTGCTCCGCCCCTAAGTCGGGAAGGTTCCTTGCGGTTCGCGGCGTGC
CGGACGTGACAAACGGAAGCCGCACGTCTCACTAGTACCCTCGCAGACGGACAGCGCC
AGGGAGCAATGGCAGCGCGCCGACCGCGATGGGCTGTGGCCAATAGCGGCTGCTCAGC
AGGGCGCGCCGAGAGCAGCGGCCGGGAAGGGGCGGTGCGGGAGGCGGGGTGTGGGGC
GGTAGTGTGGGCCCTGTTCCTGCCCGCGCGGTGTTCCGCATTCTGCAAGCCTCCGGAGC
GCACGTCGGCAGTCGGCTCCCTCGTTGACCGAATCACCGACCTCTCTCCCCAGGGATCC

mPGK:

GCAGAACCTTAATTAAAAATTCTACCGGGTAGGGGAGGCGCTTTTCCCAAGGCAGTCT
GGAGCATGCGCTTTAGCAGCCCCGCTGGGCACTTGGCGCTACACAAGTGGCCTCTGGC
CTCGCACACATTCCACATCCACCGGTAGGCGCCAACCGGCTCCGTTCTTTGGTGGCCCC
TTCGCGCCACCTTCTACTCCTCCCCTAGTCAGGAAGTTCCCCCCCGCCCCGCAGCTCGC
GTCGTGCAGGACGTGACAAATGGAAGTAGCACGTCTCACTAGTCTCGTGCAGATGGAC
AGCACCGCTGAGCAATGGAAGCGGGTAGGCCTTTGGGGCAGCGGCCAATAGCAGCTTT
GCTCCTTCGCTTTCTGGGCTCAGAGGCTGGGAAGGGGTGGGTCCGGGGGCGGGCTCAG
GGGCGGGCTCAGGGGCGGGGCGGGCGCCCGAAGGTCCTCCGGAGGCCCGGCATTCTGC
ACGCTTCAAAAGCGCACGTCTGCCGCGCTGTTCTCCTCTTCCTCATCTCCGGGCCTTTCG
ACGGATCCGGATGCAT

hFTH1:

TTAATTAATCCGCCAGAGCGCGCGAGGGCCTCCACCGGCCGCCCCTCCCCCACAGCAG
GGGCGGGGTCCCGCGCCCACCGGAAGGAGCGGGCTCGGGGCGGGCGGCGCTGATTGG
CCGGGGCGGGCCTGACGCCGACGCGGCTATAAGAGACCACAAGCGACCCGCAGGGCC
AGACGTTCTTCGCCGAGAGTCGTCGGGGTTTCCTGCTTCAACAGTGCTTGGACGGAACC
CGGCGCTCGTTCCCCACCCCGGCCGGCCGCCCATAGCCAGCCCTCCGTCACCTCTTCAC
CGCACCCTCGGACTGCCCCAAGGCCCCCGCCGCCGCTCCAGCGCCGCGCAGCCACCGC
CGCCGCCGCCGCCTCTCCTTAGTCGCCGCCGGATCC

CAG:

TTAATTAATCGACATTGATTATTGACTAGTTATTAATAGTAATCAATTACGGGGTCATT
AGTTCATAGCCCATATATGGAGTTCCGCGTTACATAACTTACGGTAAATGGCCCGCCTG
GCTGACCGCCCAACGACCCCCGCCCATTGACGTCAATAATGACGTATGTTCCCATAGTA
ACGCCAATAGGGACTTTCCATTGACGTCAATGGGTGGACTATTTACGGTAAACTGCCCA
CTTGGCAGTACATCAAGTGTATCATATGCCAAGTACGCCCCCTATTGACGTCAATGACG
GTAAATGGCCCGCCTGGCATTATGCCCAGTACATGACCTTATGGGACTTTCCTACTTGG
CAGTACATCTACGTATTAGTCATCGCTATTACCATGGGTCGAGGTGAGCCCCACGTTCT
GCTTCACTCTCCCCATCTCCCCCCCCTCCCCACCCCCAATTTTGTATTTATTTATTTTTTA
ATTATTTTGTGCAGCGATGGGGGCGGGGGGGGGGGGGGCGCGCGCCAGGCGGGGCGG

Biology Open • Supplementary information

GGCGGGGCGAGGGGCGGGGCGGGGCGAGGCGGAGAGGTGCGGCGGCAGCCAATCAG<br>AGCGGCGCGCTCCGAAAGTTTCCTTTTATGGCGAGGCGGCGGCGGCGGCGGCCCTATA<br>AAAAGCGAAGCGCGCGGCGGGCGGGAGTCGCTGCGTTGCCTTCGCCCCGTGCCCCGCT<br>CCGCGCCGCCTCGCGCCGCCCGCCCCGGCTCTGACTGACCGCGTTACTCCCACAGGTGA<br>GCGGGCGGGACGGCCCTTCTCCTCCGGGCTGTAATTAGCGCTTGGTTTAATGACGGCTC<br>GTTTCTTTTCTGTGGCTGCGTGAAAGCCTTAAAGGGCTCCGGGAGGGGGATCC
**Ly6Gopt**<br><br>GCAGAACCCCTGCAGGCCACCATGGACACCTGTCATATCGCCAAGAGCTGCGTGCTGA<br>TCCTGCTGGTGGTTCTGCTGTGTGCCGAGCGAGCACAGGGACTGGAGTGCTACAACTGT<br>ATCGGCGTGCCACCTGAGACAAGCTGCAACACCACCACCTGTCCTTTCAGCGACGGCTT<br>CTGTGTGGCCCTGGAAATCGAAGTGATCGTGGACAGCCACCGCAGCAAAGTGAAGTCC<br>AACCTGTGCCTGCCTATCTGCCCCACCACACTGGACAACACCGAGATCACAGGCAACG<br>CCGTGAACGTGAAAACCTACTGCTGCAAAGAGGACCTCTGCAACGCCGCTGTTCCAAC<br>AGGCGGAAGCTCTTGGACAATGGCTGGCGTGCTGCTGTTCAGCCTGGTGTCTGTTCTGC<br>TGCAGACCTTCCTGTGAGCATGCCAATTCTCATCGATTGCATTGGGTCGACGGATGCAT

Biology Open • Supplementary information
